# Letter from Paris

**Published:** 1854-04

**Authors:** 


THE NORTH-WESTERN
MEDICAL AND SURGICAL JOURNAL.

NEW SERIES.

VOL. III. APRIL, 1854. NO. 4.

### ORIGINAL COMMUNICATIONS.

ARTICLE I.—*Letter from Paris.*

PARIS, Feb. 13th, 1854.

DR. JOHNSON,

DEAR SIR: There are so many objects of interest in Paris, connected with the Medical Profession, that one scarcely knows which to select for the subject of a single letter.

To the stranger who was familiar with the Institutions in 1840, it does not appear, on a return at present, that much progress has been made, either in the means of teaching the doctrines taught, or the qualifications of the Professors. The Museum of the School of Medicine, comprising healthy and comparative anatomy, specimens of materia medica, surgical instruments, etc., has been principally created since that period, and although not large for a national collection, is a precious addition to the means of instruction, and contains many preparations of the nervous and lymphatic systems of unequalled beauty. This collection is due to Orfila, whose name it bears. In addition to this, a new museum has been collected at the dissecting amphitheatre of Clamart, which contains some valuable pieces, but its situation, and the rules in regard to entrance, render it useless for the purposes of instruction. None of the hospitals have collections except that of Val-de-Grace, and most of the valuable specimens which might be derived from them, are lost for want of care. The Musee Dupuytran has received considerable additions, but at the present time is inferior in every department except that of bones, which is very perfect.

The changes in the School of Medicine have not all been for the best. The death of Orfila left a void, which no attempt will be made to fill, the chair which he occupied being abolished. Dumas has also retired from teaching, and in the place of the two chairs, one of medical chemistry is to be instituted, and to be filled by Souberain, already distinguished at the School of Pharmacy. Trousseau, the unrivalled Professor of Materia Medica, has been transformed into a teacher at the Hotel Dieu, where he lectures to a small number of students, his lectures revolving around the orifice of the larynx, and scarcely ever embracing more than certain diseases of the air-passages. Andral is at present but little followed, and shows strongly the marks of advancing age, and the same may be said of Cruveilhier. On the other hand, Denonvilliers has succeeded Brechet in the chair of Anatomy, and gives a brilliant and popular course in place of that but little followed of his predecessor. Malgaigne has succeeded Blandin and his course is much admired and followed. In the Medical Clinique Bouillaud, Piorry and Rostan are still the Professors. Certain peculiarities of the first-named have so increased upon him, as to render his teaching intolerable. The second is still occupied in percussing the spleen, and marking its outlines, while Rostan continues his sound and valuable teaching in his wards at the Hotel Dieu. In the Surgical Clinique, the Professors are Roux, Velpeau, Laugier and Nelaton; the two former, too well known to need to be spoken of, but gradually declining from the effects of age; the two latter, highly popular, and rapidly advancing to the summit of reputation. The Clinique of Accouchments is directed by Paul Dubois, as formerly, than whose teaching nothing more admirable can be desired. It will be seen that in the Faculty, the gain scarcely compensates for the loss, and if the reputation of the School of Paris depended upon that alone, it might well be supposed to be upon the decline; but such is not the case, and the able corps of hospital physicians and surgeons, with the agreges chefs de clinique, internes, etc, keep up the public and private course to their former standard of excellence, although Louis has retired from his wards, and left vacant a place which cannot be filled.

Among the most notable defects of French teaching, may be noticed the neglect of general pathology and the use of the microscope. Robin is nearly the only teacher of the microscope, and his class is generally confined to a small number of American students. Nachet, one of the principal manufacturers of microscopes, told me that his best customers were from America, and that nearly all his most expensive instruments were sent there. The manufacturers of anatomical models of papier mache, plaster and wax, the preparers of skeletons and specimens of natural history, and the instrument makers, all assured me that their best customers are Americans or English. Under the head of Instruction, I should by no means forget to mention the course of Bernard, at the College of France, on Physiology; strangers, however, are apt to be disappointed in his course, as his brilliant discoveries lead them to expect too much. In other parts of his course, not relating to his own particular discoveries, he principally follows Magendie, whose place he supplies. I think that the premature publication of his opinions regarding a retrograde circulation from the veins into the arteries, through the kidneys, has tended to lessen the confidence of his hearers in his statements. His work on Experimental Physiology, which has been announced, will probably not be issued for a long time. At the side of Bernard, in the College of France, is Coste, whose lectures upon the development of organized bodies, is the most eloquent and instructive course I have ever listened to. The substance of his course is contained in his great work entitled "General and Special History of the Development of Organized Beings," which has reached its third number, being published by the government. He is known in America from his efforts in introducing the artificial breeding of fishes, a process now employed in all the different countries of Europe. I saw in his private room in the College of France, a number of troughs of earthenware, containing the eggs of fishes of the choicer kinds, such as salmon, from the lakes of Switzerland, and other kinds from the different rivers of Europe. Prof. Coste informed me that the eggs are transported without difficulty, a distance of 800 miles by land, and that they could easily be sent to America without injury. These eggs are of the size of peas, of a bright red color, and to be hatched are placed upon little racks made of willow twigs, which are fixed in the troughs so as to be covered with water, and a gentle current is kept playing over them. I saw them in every different stage of development, from the simple egg to the fish just bursting from it. It is a curious sight, and seems to promise much benefit to man, by supplying fresh and wholesome food. It has, however, its difficulties. Like other orphans, a large portion of these fish brought up by hand, die before attaining maturity, and it is yet doubtful whether the plan will be successful, excepting with regard to those fishes found in the waters where the breeding is carried on; in this case there seems no reason to doubt its full success.

An interesting fact has incidentally been noticed by those engaged in observing this process of hatching; it is that a great number of monsters occur amongst the newly-hatched fishes, a number much larger in proportion than occurs in the mammalia.

In speaking of the scientific men of Paris, you will naturally expect me to say something of Lebert: he does not reside here at present but holds a professorship in a German University. His great work, however, on which he has been engaged for many years, is about to be put to press, and it is confidently expected that it will be by far the most perfect treatise on morbid anatomy which has ever been published. Bailliere, the great publisher, showed me some of the drawings which are in his hands to be engraved, and they are indeed magnificent. The work is so expensive that it was difficult to find a publisher to undertake it, but as the Minister of Public Instruction advances a certain sum towards the expense, and the Prussian government subscribes for a certain number of copies, Bailliere was induced to undertake it. The first number will be issued soon. While speaking of forthcoming works, I may mention that Guerin has one in hand and far advanced, on the subject of deformities, which is to be illustrated in the most perfect manner by beautifully-executed and colored plates. M. Guerin has devoted a great part of his life, and a considerable sum of money to this object, and has collected a museum comprising about 3000 pieces, illustrating every different point of the subject. This collection, which may be regarded as a national one, is now offered for sale, and it were greatly to be desired that it should be transferred to some institution in America; as may be supposed, from the labor and money expended upon it, the price at which it is valued is not small. It may be interesting to some of your readers to know that M. Guerin, who was appointed by the Academy of Medicine some years since, to make a report on the subject of cholera, has nearly accomplished his task, and is about to submit it. After the fullest investigation and the examination of innumerable documents and publications of every kind, he assures me that he is fully convinced of its communicability, but only in certain conditions, and in a manner peculiar to itself. I think this opinion is beginning to have great influence here, since the cholera patients, which were at first carried into all the wards, have lately had particular halls assigned to them, separated as much as possible from the others. So long as they were carried into the general wards, the number of cases regularly increased, but so soon as the change was made, they diminished. As this is a doctrine which I had advocated for many years, it is gratifying to find its truth at length being recognized, more particularly as it is the only means by which the spread of the disease can be explained, and its ravages, to a certain extent, controlled. If time and space permitted, I should still have much to say of societies, journals, medical doctrines and men, past and present, but as I have occupied enough of both, they must be deferred to another time, or entirely omitted.

Very truly yours, B.

